# Pairwise protein expression classifier for candidate biomarker discovery for early detection of human disease prognosis

**DOI:** 10.1186/1471-2105-13-191

**Published:** 2012-08-07

**Authors:** Parminder Kaur, Daniela Schlatzer, Kenneth Cooke, Mark R Chance

**Affiliations:** 1 Case Center for Proteomics and Bioinformatics, Case Western Reserve University, Cleveland, OH 44106, USA; 2 Case Center for Proteomics and Bioinformatics, Case Western Reserve University, Cleveland, OH 44106, USA; 3 Pediatric Hematology and Oncology, University Hospitals, Cleveland, OH 44106, USA; 4 Case Center for Proteomics and Bioinformatics, Case Western Reserve University, Cleveland, OH 44106, USA

**Keywords:** Biomarker discovery, Idiopathic Pneumonia Syndrome, Classification, ProtPair classifier.

## Abstract

**Background:**

An approach to molecular classification based on the comparative expression of protein pairs is presented. The method overcomes some of the present limitations in using peptide intensity data for class prediction for problems such as the detection of a disease, disease prognosis, or for predicting treatment response. Data analysis is particularly challenging in these situations due to sample size (typically tens) being much smaller than the large number of peptides (typically thousands). Methods based upon high dimensional statistical models, machine learning or other complex classifiers generate decisions which may be very accurate but can be complex and difficult to interpret in simple or biologically meaningful terms. A classification scheme, called ProtPair, is presented that generates simple decision rules leading to accurate classification which is based on measurement of very few proteins and requires only relative expression values, providing specific targeted hypotheses suitable for straightforward validation.

**Results:**

ProtPair has been tested against clinical data from 21 patients following a bone marrow transplant, 13 of which progress to idiopathic pneumonia syndrome (IPS). The approach combines multiple peptide pairs originating from the same set of proteins, with each unique peptide pair providing an independent measure of discriminatory power. The prediction rate of the ProtPair for IPS study as measured by leave-one-out CV is 69.1%, which can be very beneficial for clinical diagnosis as it may flag patients in need of closer monitoring. The “top ranked” proteins provided by ProtPair are known to be associated with the biological processes and pathways intimately associated with known IPS biology based on mouse models.

**Conclusions:**

An approach to biomarker discovery, called ProtPair, is presented. ProtPair is based on the differential expression of pairs of peptides and the associated proteins. Using mass spectrometry data from “bottom up” proteomics methods, functionally related proteins/peptide pairs exhibiting co-ordinated changes expression profile are discovered, which represent a signature for patients progressing to various disease conditions. The method has been tested against clinical data from patients progressing to idiopthatic pneumonia syndrome (IPS) following a bone marrow transplant. The data indicates that patients with improper regulation in the concentration of specific acute phase response proteins at the time of bone marrow transplant are highly likely to develop IPS within few weeks. The results lead to a specific set of protein pairs that can be efficiently verified by investigating the pairwise abundance change in independent cohorts using ELISA or targeted mass spectrometry techniques. This generalized classifier can be extended to other clinical problems in a variety of contexts.

## Background

A biomarker is an indicator of a specific biological condition, such as presence or progression of a disease, or treatment response of a drug. Studying protein expression data to assess utility as potential biomarkers holds special significance since cellular behavior and disease are functions of the abundance and interactions between proteins involved in biological phenomenon [1]. The expression of specific proteins are a function of disease state, prognosis and recovery, and comparative proteomics holds special promise for revealing such candidates. Tremendous efforts are being made to find novel biomarkers in a wide variety of fields including cancer research [2], cardiovascular disease [3], kidney disease [4], and neurodegenerative complications [5]. Reliable proteomics biomarkers are urgently needed to identify and target the patients that are likely to progress to a disease for treatment intervention earlier in the course of the disease as well as to identify patients that are unlikely to progress, and for the evaluation of therapeutic response. However, progress so far has been slow.

Mass spectrometry is increasingly used for relative or absolute quantification of peptides and proteins. Techniques such as stable isotope labeling, spectral counting, and spectral feature analysis have particularly accelerated growth in the field of quantitative proteomics [6-10]. Challenges associated with analyzing data originating from proteomic biomarker discovery experiments share similarity with transcriptional profiling, such as the interpretation of complex biological samples and the statistical inference associated with high-dimensionality data sets resulting from a much smaller number of samples compared to the variable number of analytes [11-13]. Determining the buried structure within such data reliably, such as correlation coefficient or higher-dimensional patterns, is highly difficult in this limited sample situation. Given the challenges in computational modeling with limited sample size and model complexity, some simplifying assumptions (such as reducing the dimensionality of the data or the family of classifiers) are typically made [13]. There is a dilemma associated with some of the current methods routinely used for proteomics data analysis since it can be difficult to derive biologically relevant conclusions from the highly complex non-linear decision boundaries between classes of interest resulting from some of the standardized pattern recognition tools such as neural networks [14], decision trees [15], and support vector machines [16]. Pairwise expression analysis has been used successfully in the 2 dimesional gel electrophoresis and mRNA expression profiling studies [17,18]. Using pairs of peptide markers in MS based proteomics studies, as opposed to the single markers used in traditional analysis, allows for separation to be made in a 2-dimensional space, allowing for possibly greater discriminability arising from a greater (twice) amount of information used in making a decision. Existing evidence [13,19,20] indicates that simpler classification methods [21] exhibit comparable performance to that of more complex models for such cases. In this study, we investigate the value of such relatively simple classification schemes in the context of proteomics experiments to extend the well documented advantages observed for transcriptional profiling classification to proteomics biomarker discovery. Our results support and extend this finding.

We present a simple comparison-based approach to classifying protein expression profiles, the ProtPair classifier, that first differentiates patients according to pre-defined clinical variables by finding pairs of peptides whose relative expression levels change significantly from one condition to the other. Second, each peptide is then mapped to its protein identity, and peptide pairs originating from the same pair of proteins are grouped together to form protein pairs. Lastly, the resulting protein pairs are investigated for their ability to consistently differentiate between the two clinical states across all possible peptide pairs, the extent of protein coverage exhibiting the differential expression, and consistency in the direction of change to arrive at the final protein pair that leads to the best classification. Multiple peptide pairs originating from the same pairs of proteins indicating similar patterns of expression change lead to the top scoring protein pair candidates for the clinical problem under consideration. The method does not rely on data normalization, relies on a pair of variables that are reproducibly observed, is suitable to work with small training data sets, and can provide biologically meaningful biomarkers.

Rank-based approaches to gene pair selection and classification for classifying gene expression profiles from pairwise mRNA expressions have been successfully employed [19,20,22-24]. The full potential of such methods for proteomics can now be realized as high-throughput protein comparisons with ascertainment of thousands of peptides are routine using mass spectrometry based proteomics [25]. Making predictions based upon relative concentrations of proteins rather than genes provides a natural and stronger link with biochemical activity. We hope to realize those benefits towards characterizing proteomics experiments through modified approaches tailored specifically for such studies. ProtPair generates specific hypothesis for follow-up studies, employs few proteins for classification and is easy to interpret. Our approach to selecting informative pairs of proteins is an attempt to exploit additional information gained from capturing such joint statistics, with perturbations in pairwise expressions potentially resulting from protein-protein interactions in extended networks.

The efficacy of ProtPair is demonstrated on a clinical proteomics dataset involving patients progressing to Idiopathic Pneumonia Syndrome (IPS) following stem cell transplantation (SCT) [26,27]. The primary treatment option available for patients diagnosed with certain malignant and non malignant diseases is allogeneic hematopoietic SCT. However, success of the procedure is limited due to a number of complications arising following the intervention. Idiopathic pneumonia syndrome (IPS) is an occasionally observed alveolar injury following SCT without the presence of an active lower respiratory tract infection. Depending upon the bone marrow donor, IPS can be manifested in 5-15% of patients. IPS typically begins its onset after about 18 days following the SCT, with a mortality rate of >70%. Molecular biomarkers with even modest predictive power to predict disease progression would have very high clinical value. Existing approaches to study IPS lack comprehensiveness as they target only a few known inflammatory proteins, and thus, are limited with respect to expanding the disease pathway. In this study, discovery-based, quantitative proteomics is utilized to provide the identification and quantification of hundreds of proteins across global plasma proteome in an unbiased, comprehensive manner. ProtPair is used to uncover robust markers that differentiate patients that progress to IPS from non-progressors, so that targeted individualized therapy can be designed for SCT related complications.

## Methods

Blood specimens were obtained at the time of stem cell transplantation (Day 0). Plasma was separated from the samples and stored at -80°C until analysis. Aliquots for individual plasma samples were thawed and depleted of the seven most abundant proteins. Bovine trypsin was used for proteolytic digestion. Six hundred nanograms of each sample were analyzed by liquid chromatography coupled with mass spectrometry using LTQ-FT as previously described [28], and the order of sample injections was randomized over all samples. The chromatograms across all the spectra thus obtained were time aligned across multiple runs using Rosetta Elucidator (Rosetta Biosoftware, Seattle, WA) [29,30]. Proteins were identified using Mascot and protein teller within Rosetta Elucidator framework [29-33], raw peak areas corresponding to each peptide were used for peptide quantification, and were calculated from the selected ion chromatograms (SICs) using Rosetta Elucidator. No normalization/scaling was performed and peak areas were directly used for ProtPair analysis. A total of 21 patients were investigated using 2 technical replicates from each patient. Thirteen of the patients developed IPS following the transplants, while eight patients remained unaffected. The outcome under study was classification of the SCT patients into those who subsequently developed IPS or those who remained unaffected by complications. Protein pairs assigned high significance by ProtPair were imported into Ingenuity Pathway Analysis (IPA) (Ingenuity Systems, Redwood City, CA, USA) to uncover protein networks enriched in the candidate proteins. The software generates networks based upon biomedical literature and existing protein interaction databases to reveal biological networks associated with the candidate proteins.

All patients (or their surrogates) and controls gave written, informed consent in accordance with the Declaration of Helsinki and the trial was approved by the respective Institutional Review Boards of the University of Michigan and the Dana-Farber Cancer Center.

### Algorithm Description

Consider that the two clinical states to be distinguished are: patients that progress to a certain disease, called “progressors” (labeled *P*); and patients who are unaffected by the disease, called “controls” (labeled *C*) after a certain course of time. Let *p*_1_, *p*_2_, *p*_3_,…,*p*_*N*_ represent the peptides identified across all clinical samples. Let $I_{C_{\text{ik}}}$ and $I_{P_{\text{ik}}}$ represent intensities of peptide *i* obtained from *k*^*th*^control and *k*^*th*^progressor patient respectively. The intensity values are calculated by integrating the peak area under the SIC with a 10 ppm window for the corresponding peptide using Rosetta Elucidator, and no pre-processing/transformation is performed on the peak areas in order to minimize artifacts that may result from such processing. All possible pairs of detected peptides are generated and examined. If there are N number of detected peptides within a sample across all experiments, they’ll constitute $\left(\begin{array}{c} N\\ \;2 \end{array}\;\right)$unique pairs of peptides where $\left(\begin{array}{c} N\\ \;2 \end{array}\;\right)=\frac{N!}{2!(N-2)!}$, where ! denotes factorial operation (*N*!=*N*×(*N*−1)×(*N*−2)×…3×2×1). In order to compare the relative abundance, the ratio of intensities of peptide pair (*i*,*j*) arising from peptides *p*_*i*_ and *p*_*j*_ for *k*^*th*^ patient for controls and progressors respectively is evaluated as follows:

(1) $$R_{C_{\text{ijk}}}=\frac{I_{C_{\text{ik}}}}{I_{C_{\text{jk}}}},R_{P_{\text{ijk}}}=\frac{I_{P_{\text{ik}}}}{I_{P_{\text{jk}}}}$$

The goal of ProtPair is to find a pair of peptides/proteins that is the most discriminative between the two clinical conditions of interest. The discriminative power of a peptide pair (*ij*) is estimated by its Discriminability Index (DI), *d*_*ij*_, and is defined as under [34]:

(2) $$d_{\text{ij}}=\frac{\mu_{\frac{1}{2}}(R_{C_{\text{ij}}})-\mu_{\frac{1}{2}}(R_{P_{\text{ij}}})}{\sqrt{\sigma_{R_{C_{\text{ij}}}}\times \sigma_{R_{P_{\text{ij}}}}}}$$

where $\mu_{\frac{1}{2}}$ and *σ*symbolize the median and standard deviation of the input argument, which represent peptide pair intensity ratios for all controls and progressors as defined in equation 1. The median value for the ratios indicates the central tendency of the data and is robust in the presence of outlier values, whereas standard deviation measures the variation within the peptide ratio relative values for the two clinical categories. The discriminability index is a measure of the distance between the two relative abundance distributions among the controls versus the progressors. It describes the inherent and unchangeable properties arising from the two distributions and hence assists in selecting the most discriminative features (peptide pairs in this case), and is independent of the decision strategy employed. Discriminability is increased either by increasing the separation (numerator) or by decreasing the spread (denominator) of individual ratio distributions. The most discriminative peptide pair is determined as follows:

(3) $$\left(m,n)=\text{arg}\underset{i,j}{\text{max}}|d(i,j)\right|$$

Thus, the peptide pair providing the highest absolute value of DI among all possibilities is assigned to be the highest scoring pair.

Each of the peptides is assigned protein identity using Rosetta Elucidator (Rosetta Biosoftware, Seattle, WA), peptides that correspond to more than a single protein in the sequence database are allocated among all corresponding proteins, and a minimal protein list sufficient to account for the observed peptide assignments is derived using the expectation maximization algorithm [32]. For further increasing the confidence of protein ID assignment, only proteins identified with a minimum of 2 high scoring peptide assignments were considered for candidacy by Protpair. As proteins act as the true molecular functional units, and are likely to be affected during a disease, we seek to find protein pairs that best classify the two clinical states. This is done by first generating the list of all possible protein pairs. If there are *M* number of proteins detected across all experiments, there will be $\left(\begin{array}{c} M\\ \;2 \end{array}\;\right)$protein pairs. The resulting protein pairs are investigated for their ability to consistently differentiate between the two clinical states across all possible peptide pairs, the extent of protein coverage exhibiting the differential expression, and consistency in the direction of change to arrive at the final protein pair that leads to the best classification. DI for a protein pair P1 and P2 is calculated as follows:

(4) $$D(P1,P2)=\mu_{\frac{1}{2}}(d(i,j))\;\;\forall p_{i}\in P1,p_{j}\in P2$$

Here, ∀ denotes “for all”, and ∈ “belongs to”. For example, ∀*p*_*i*_∈*P*1 means - for all the peptides orginating from protein P1. Thus, the DI factor for proteins *P1* and *P2* is defined to be the median value of the DI across all peptide pairs from proteins *P1* and *P2*. The highest ranked protein pair is obtained by the following equation:

(5) $$\left(m,n)=\text{arg}\underset{i,j}{\text{max}}|D(i,j)\right|$$

This means that the candidate pair providing the highest median value of DI across all peptide possibilities is assigned to be the top ranked protein pair.

## Results and discussion

### Results

The overall analysis of mass spectrometry data using Mascot within Rosetta Elucidator framework identified 1799 peptides resulting from 151 unique proteins across all patient samples. In order to allow for confident identification, a false discovery rate of 1% was used as a threshold for the identification of peptides. A total of 112 out of the 151 identified proteins which were found to be identified by at least 2 or more high scoring peptides, were further examined by ProtPair, and the rest of 39 peptides were excluded from examination. After eliminating single peptide hits for a protein, 1760 peptides were remaining, which were taken as pairs (leading to 1547920 unique peptide pairs) in order to determine their discriminability. The resulting peptide pairs were further grouped into the corresponding 6216 unique protein pairs (formed from 112 proteins, taken two at a time), and the discriminabilty of the pairwise proteins was investigated.

Table 1 shows the list of protein pairs ranked by their DI score. If proteins in columns 1 and 2 are denoted by P1 and P2 respectively, the ratio of intensities of P1:P2 is higher in the case of IPS progressor patients than that for controls. This implies that in the case of IPS progressors, either (a) P1 is upregulated, or (b) P2 is downregulated, or (c) both (a) and (b) are true. Column 4 denotes the p-value (probability that a particular score would occur by chance) associated with each DI score. Since multiple hypotheses (6216) corresponding to each protein pair are being tested, false discovery rate (FDR) was calculated (Column 5) using empirical null model from permutation tests in order to correct for multiple hypothesis testing [35-38]. For a specified DI score threshold, say, T, the number *S*_*true*_ of observed scores ≥ T and the number *S*_*null*_of null scores ≥ T are counted. Assuming that the total number of observed scores and null scores are equal, then the estimated FDR is simply $\frac{S_{\text{null}}}{S_{\text{true}}}$.

**Table 1:** Protein pairs ranked by the discriminability index scores

**Protein 1 (upregulated in IPS progressors)**	**Protein 2 (downregulated in IPS progressors)**	**DI score**	**p-value**	**FDR rate**
APCS Serum amyloid P-component	HGFAC Hepatocyte growth factor activator	2.01	0	0.00
C8G Complement component C8 gamma chain	HGFAC Hepatocyte growth factor activator	1.60	1.6×10^−6^	0.01
APCS Serum amyloid P-component	CFHR1;LOC100293069 Complement factor H-related 1	1.58	1.6×10^−6^	0.01
C4BPA C4b-binding protein alpha chain	ENO2 Gamma-enolase	1.48	6.3×10^−6^	0.01
C4BPA C4b-binding protein alpha chain	HGFAC Hepatocyte growth factor activator	1.45	9.5×10^−6^	0.01
APOA4 Apolipoprotein A-IV	ALB Putative uncharacterized protein ALB	1.44	9.5×10^−6^	0.01
APCS Serum amyloid P-component	CFHR2 Isoform Short of Complement factor H-related protein 2	1.43	1.1×10^−6^	0.01
F2 Prothrombin (Fragment)	HGFAC Hepatocyte growth factor activator	1.37	3.5×10^−6^	0.03
APOD Apolipoprotein D	HGFAC Hepatocyte growth factor activator	1.35	5.7×10^−6^	0.05
CPB2 Isoform 1 of Carboxypeptidase B2	HGFAC Hepatocyte growth factor activator	1.31	9.3×10^−6^	0.08
SERPINA6 Corticosteroid-binding globulin	HGFAC Hepatocyte growth factor activator	1.30	9.8×10^−6^	0.09
APCS Serum amyloid P-component	F10 Coagulation factor X	1.30	1.1×10^−4^	0.09
C8A Complement component C8 alpha chain	HGFAC Hepatocyte growth factor activator	1.26	1.6×10^−4^	0.11
APOA4 Apolipoprotein A-IV	GPX3 Glutathione peroxidase 3	1.25	1.7×10^−4^	0.10
APOB Apolipoprotein B-100	HGFAC Hepatocyte growth factor activator	1.24	1.8×10^−4^	0.10
FGG Isoform Gamma-B of Fibrinogen gamma chain	AZGP1 alpha-2-glycoprotein 1, zinc”	1.23	2.0×10^−4^	0.10
AGT Angiotensinogen	HGFAC Hepatocyte growth factor activator	1.22	2.1×10^−4^	0.10
APCS Serum amyloid P-component	ENO2 Gamma-enolase	1.18	2.8×10^−4^	0.13
CPB2 Isoform 1 of Carboxypeptidase B2	C1QC Complement C1q subcomponent subunit C	1.17	2.9×10^−4^	0.12
APCS Serum amyloid P-component	GPX3 Glutathione peroxidase 3	1.17	3.0×10^−4^	0.11
C4BPA C4b-binding protein alpha chain	LBP Lipopolysaccharide-binding protein	1.16	3.0×10^−4^	0.11
APCS Serum amyloid P-component	LOC653879 similar to complement component 3	1.15	3.3×10^−4^	0.11
CPB2 Isoform 1 of Carboxypeptidase B2	AFM Afamin	1.15	3.3×10^−4^	0.10

**Figure 1:**
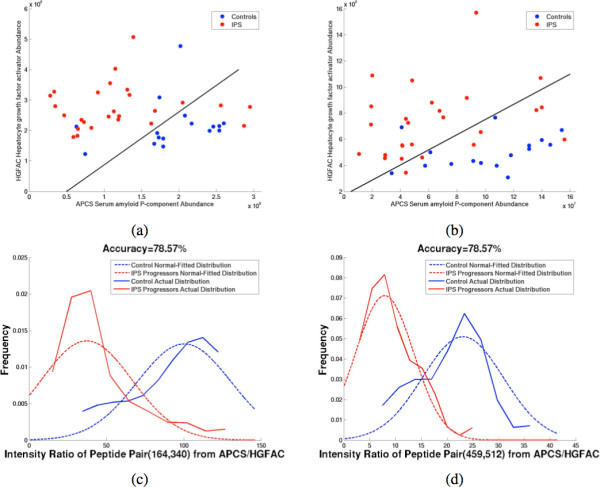
**(a) and (b) Scatter plots for two pairs of peptides from top protein pair APCS (Serum amyloid P-component) and HGFAC (Hepatocyte growth factor activator).** The two classes are represented using red and blue, the axes represent the abundance levels of the two peptides and the black line represents the decision boundary. Peptide sequences of APCS in Figure 1a and 1b: GYVIIKPLVWV, DNELLVYK, while corresponding sequences for HGFAC: LCNIEPDER and LHKPGVYTR. **(c)** and **(d)** Distribution of peptide signal abundance ratios ( $\frac{\text{APCS}}{\text{HGFAC}}$) from two unique peptide pairs originating from proteins APCS and HGFAC. Red and Blue indicate control and IPS progressors respectively.

Figures 1a and 1b show the scatter plot of two unique peptide pair abundances from top scoring protein pair (Table 1) APCS (Serum amyloid P-component) [39] and HGFAC (Hepatocyte growth factor activator) [40], indicating higher levels of HGFAC in the IPS progressors (red dots) as opposed to controls (blue dots), while levels of APCS tend to be lower in those cases. Figures 1c and 1d illustrate the distribution of ratios of intensity signals from two of the most discriminative peptide pairs originating from proteins APCS and HGFAC. The blue lines indicate the control samples, while red lines represent the IPS progressors. The solid lines indicate the true distribution of patients, while the dotted lines are the best fitting corresponding Gaussian distributions.In both cases, the measurements independently suggest the overall trend that APCS is downregulated while HGFAC is upregulated in patients that progress to IPS, and is vice-versa for control samples. The same trend of differential expression of proteins is observed across all other peptide pair possibilities as indicated by their DI values leading to the highest median value across all protein pairs.

The top scoring protein pair APCS and HGFAC provided highest discriminability across all peptide pairs, APCS is an acute phase response protein whose concentration is known to change significantly in response to inflammation [39,40]. Thus, the data suggests that patients with dysregulation in the concentration of specific acute phase response proteins at the time of bone marrow transplant are highly likely to develop IPS within 2-3 weeks.

**Figure 2:**
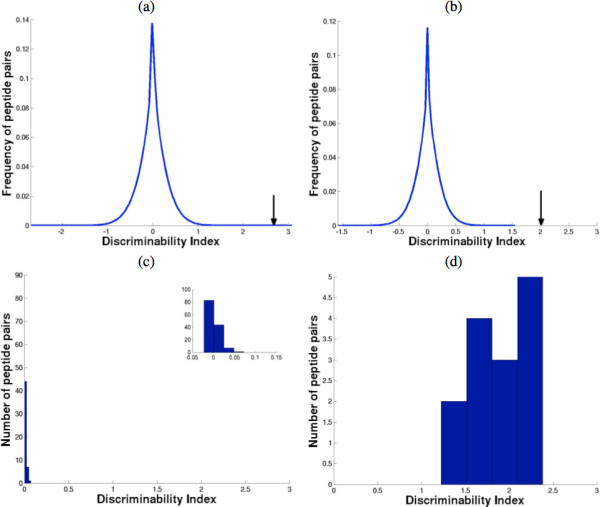
(a) DI score distribution of peptide pairs with random permutation of class labels, the location of true highest scoring peptide pair is indicated by arrow (b) Median DI score distribution for protein pairs with randomly assigned class labels, with true top scoring protein pair shown using arrow (c) DI score distribution from a randomly picked protein pair across all constituent peptides (d) DI score distribution from the highest scoring protein pair, APCS and HGFAC.

Figures 2a and 2b depict the significance of the DI scores assessed using permutation tests for peptide and protein pairs respectively. Figure 2a represents null distribution of the DI for peptide pairs generated using a total of 100 random permutations of class labels, while maintaining the original sample size for individual clinical category. During each permutation, DI score for all peptide pairs is calculated, and Figure 2a shows the distribution of DI from all permutations. The true top peptide score obtained using “true labels” is indicated by black arrow, demonstrating that the probability of obtaining the true score from null distribution is extremely low (p-value<1.5×10^−7^). Similarly, Figure 2b represents distribution representing the median DI of the all protein pairs during 100 permutations, with arrow indicating the true top DI score using “true labels”, indicating high statistical significance of the true score (p-value<10^−20^). Note that combining multiple peptide pairs from same two protein results in highly significant scores, since its is unlikely that multiple corresponding peptide pairs show consistent, high discrimination purely “by chance”, as indicated by the low p-values and low false discovery rates in Table 1. Note that the DI scores for most of the peptide and protein pairs is centered around zero indicating that no discrimination is being provided by the such pairs. This is to be expected with permuted labels, since most proteins/peptides should not exhibit any differential expression among the arbitrary categories defined by random labels.

Figure 2c shows the DI scores from constituent peptides of a randomly picked protein pair (APOH Beta-2-glycoprotein 1 and FN1 Isoform 1 of Fibronectin), indicating that the discriminability is close to zero across all peptide pair possibilities. Apolipoprotein H (APOH) is a lipid binding protein implicated in physiologic pathways for lipoprotein metabolism, coagulation, and the production of antiphospholipid autoantibodies, while Fibronectin is involved in cell adhesion and migration processes including embryogenesis, wound healing, blood coagulation, host defense, and metastasis. The expression levels of peptide pairs from APOH and Fibronectin do not appear to affect the prognosis of IPS as indicated by the minimal discriminability seen in Figure 2c. Figure 2d depicts that the distribution of DI values of the highest scoring protein pair (APCS ang HGFAC) is significantly shifted to the right, illustrating that DI scores are consistently and significantly higher for discriminating protein pairs than their randomly chosen counterparts in Figure 2c.

**Figure 3:**
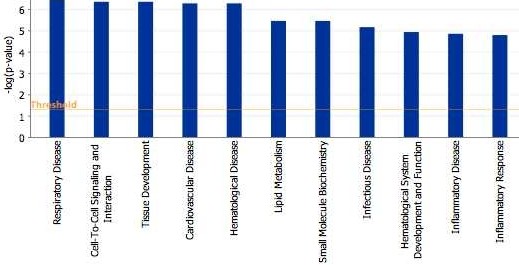
Ingenuity Pathway Analysis (IPA) analysis: Biological processes and diseases most significantly associated with top 20 proteins identified by ProtPair.

**Figure 4:**
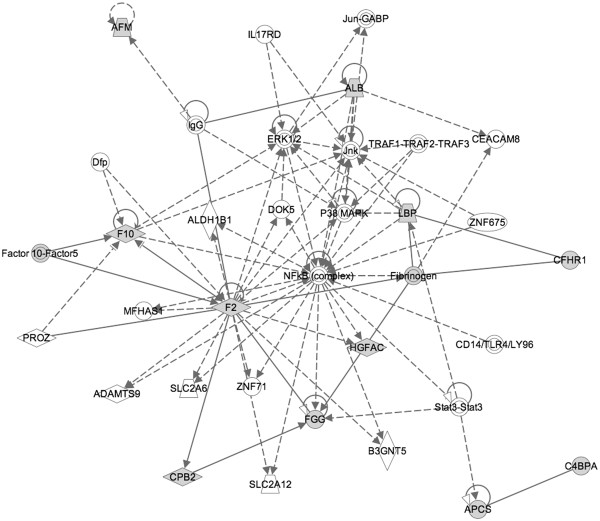
Top scoring biological network obtained using IPA analysis representing cluster of highly significant proteins identified by ProtPair.

As described in the Methods section, the proteins from top 20 pairs were imported into IPA to see if they shared common biological networks. Figure 3 shows that the network is associated with respiratory disorders, hematological dysfunction, cardiovascular complications, and infectious diseases. Figure 4 illustrates the top scoring network uncovered using IPA software, revealing the interactions between the top candidate proteins identified by ProtPair.

### Discussion

A ranking/classification methodology for biomarker discovery using pairs of proteins from shotgun mass spectrometry based proteomics data has been introduced. The method leads to concrete hypotheses about the predictive significance of specific protein expression comparisons, which can be followed up for future validation. The method has been explored using clinical proteomics data from 21 patients (8 controls, 13 disease progressors) differentiating between patients progressing to IPS versus controls that remain unaffected by IPS following the bone marrow transplant procedure. Although sample size of 21 may appear small, it is well accepted an appropriate size for the discovery and qualification phase of development of biomarker discovery [1].

**Figure 5:**
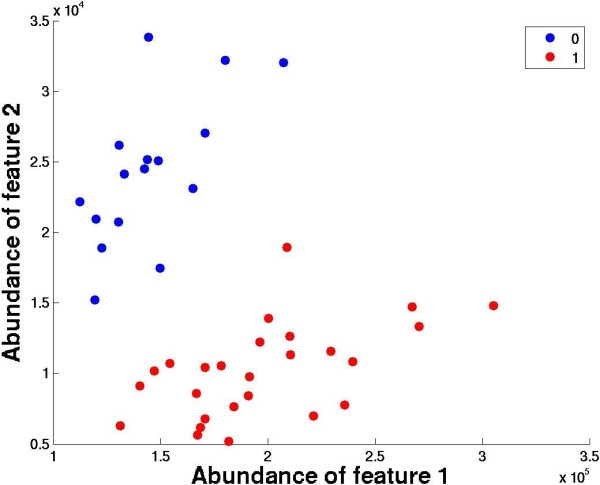
Most discriminating feature pair based upon MS1 only features.

The initial concept behind ProtPair was inspired by a rank-based approach for molecular classification based upon pairwise mRNA expression comparisons. Geman *et al* introduced top-scoring pair(s) (TSP) classifier for class prediction in which the mRNA expression levels of genes are directly compared against each other to each other to make classification [19]. The decision is thus dependent on only the following question: is the expression of gene A higher than the expression of gene B in the sample? If so, the diagnosis is class 1. If the expression of gene B is higher than for gene A, then the diagnosis is for class 2. Such decision rules implicitly draw the decision boundary line at *y*=*x*. Although this approach has been highly successful for mRNA expression experiments, our initial attempts to directly adopt this method were unsuccessful. Since peptides signals exhibit a wide dynamic range, we needed a more general approach than *y*=*x* boundary line used in mRNA studies. For example, Figure 5 shows a scenario where the two disease classes (represented by red and blue) are clearly separable, but *y*=*x*boundary line is not an optimal separation line. Thus, we propose a more general scheme without any implicit normalization assumtions.

Figures 1a and 1b show the scatter plots of peptide intensities from the top scoring proteins APCS and HGFAC. Note that as in the case of single protein/peptide markers, none of the proteins/peptides is able to provide good separation between the two categories by itself. The best way of discriminating among the two cases is by drawing a line (black, separation boundary) that is close to the “diagonal” line (suggesting that both candidates contribute towards discrimination), instead of being closer to a horizontal or a vertical line (indicating that the separation could be provided by a single protein). Hence, the combination of pairwise markers gains strength by combining the discrimination power achieved by both markers, which is not possible by treating peptide/protein markers in isolation, as is often done in the traditional analysis. When all constituent peptide pairs from two proteins exhibit consistent trend of significant differential expression change, all corresponding DI index values increase, leading to a high median DI index value across the protein pair.

The significance of the score was assessed using permutation analysis. Artificial data set was constructed by randomly rearranging class labels, while maintaining the original sample sizes of 8 and 13 for controls and IPS progressors respectively. The resulting scores mirror those obtained by pure chance without any meaningful biological significance, while maintaining the overall statistical dependency structure among peptides/proteins. Figure 2a displays the distribution of peptide pair scores obtained from 100 permutations. From this null distribution, a p-value associated with a given score from unpermuted “true” data can be computed by taking the fraction of permuted data sets in which a score of at least as large is obtained. This p-value is a measure of probability of observing a given score under the null hypothesis that the pairs are non informative for classification. Only 23 among the 154792000 (100 permutations, with 1547920 peptide pair scores in each permutation) scores came greater than or equal to the actual top score, yielding a p-value of 1.5×10^−7^. Similarly, Figure 2b shows the distribution of top scores obtained using 100 permutations from protein pairs by taking the median of all constituting peptides. No score from 6216 protein pairs among the 100 permutations came near the true top score (marked by arrow in Figure 2b), yielding a p-value of virtually 0. The method gains significant strength by combining multiple peptide pairs originating from the same set of proteins, since each unique peptide pair provides an independent measure of discriminatory power, which is unlikely to arrive by pure chance alone.

The expected generalization error rate of ProtPair for the IPS data set was performed using leave-one-out cross validation (CV). This involves using both technical replicates from a single patient as the test data, and the remaining samples as the training data, and is repeated such that samples from each patient is used exactly once as the test data. Note that in order to obtain an unbiased validation, leave one out cross validation study refers to each patient as opposed to each measurement, and all measurements from a single patient were left out during each training step. In particular, both the actual top scoring DI value, as well as the set of pairs which achieve it, can vary with the sample left out. The estimated prediction rate is $1-\frac{e}{N}$ where *e* is the number of misclassifications observed on the test data during the cross-validation, and N (42 for the case of IPS study) being the total number of samples. For this procedure, there is only a single parameter, threshold of peptide abundance ratios, to select inside the cross validation loop. For other procedures that do require multiple parameters, such as k-nearest neighbors, random forests and support vector machines, the estimated prediction rates may be highly biased if performance is sensitive to these parameters and they are not properly cross-validated. The prediction rate of the ProtPair for IPS study as measured by leave-one-out CV is 69.1% (corresponding to 13 errors across 42 samples). This is less than the 78% error rate seen in Figure 1, but is not unreasonable. In fact, a 70% prediction rate may be highly beneficial for clinical diagnosis as it may flag patients in need of more frequent monitoring. In addition, the results obtained are consistent with recent studies by more conventional statistical methods [28]. This biomarker prediction is based on data from the day of SCT, the indicated biomarkers may be altered further during progression to IPS. Note also that the CV accuracy of a classifier is highly dependent upon the biological complexity of a given disease, sample size and population diversity, and heterogeneity of the underlying phenomenon. In addition, the highest scoring pair may change when the training data is even slightly perturbed by adding or deleting a few samples so that the CV accuracy is not necessarily reflective of global accuracy provided by the ultimate final pair (HGFAC and APCS in this case). As seen in Figure 1, although there is some amount of overlap, the final protein pair consistently shows strong evidence of discrimination among the two patient populations, illustrating the emergent behavior of protein expression change across the two populations. In addition to being a classifier, ProtPair has high utility as a predictive and actionable tool to rank proteins in the order of discrimination providing candidates for future validation testing specific hypotheses.

To understand if the totality of the expressed protein list is consistent with known pathways of IPS, we used Ingenuity Pathway Analysis (IPA) to explore dysregulated pathways suggested by the proteins seen to be significantly changing. As described in the Methods section, the top 20 proteins were imported into IPA. IPA has several algorithms to identify if the set of proteins imported are associated with specific “canonical” pathways or biological processes and diseases. Nodes within the network are displayed using various shapes that represent the functional class of the protein. All edges are supported by at least one reference from the literature, from a textbook, or from canonical information stored in the Ingenuity Knowledge Base. In terms of processes and diseases (Figure 3), the imported protein set was most significantly associated with the following: respiratory disease, cell to cell signaling and interaction, tissue development, cardiovascular disease, and hematological disease (all with p values <10^−6^). Inflammatory diseases and inflammatory response were also highly significant (p values =10^−6^). In terms of canonical processes, acute phase response was top ranked (data not shown, p value <10^−7^). These results are quite encouraging and validate the method as ProtPair “top ranked” proteins are associated with the biological processes and pathways intimately associated with known IPS biology based on both mouse models and human studies, such as respiratory disease, inflammatory responses, and acute phase response [41-45]. These pathway dysregulations are consistent with recent studies [28].

In order to more specifically examine the dysregulated protein networks suggested by the top 20 proteins, we used the IPA database to create a dense sub-network of targets, where the dysregulated proteins (nodes) are shaded and IPA inserts additional nodes (colored white) and annotated interactions (edges) in order to connect as many of the targets as possible while restricting the total number of nodes added (Figure 4). For example, ProtPair identified lipopolysaccharide binding protein (LBP), Fibrinogen, Hepatocyte growth factor activator (HGFAC, a top scoring protein), fibrinogen gamma chain (FGG), coagulation factor X (F10), and thrombin (F2) as changing in case versus control samples. The changes seen for LBP have been recently confirmed by ELISA studies [28]. IPA inserted extracellular-signal-regulated kinase (ERK1/2) and nuclear factor kappa-light-chain-enhancer of activated B cells (NF-KB) as they have direct connections (e.g. annotations of binding or regulatory control) associated with those proteins. This is consistent with the overall dysregulatory themes of acute phase response and cell-cell signaling as NF-KB is transcription factor controlling expression for many acute phase response proteins and is regulated by TNF-*α*, which is a target for IPS therapies in clinical trials [43-46] while ERK1/2 proteins (also termed MAP kinases) regulate cell growth.

In its present form, ProtPair makes predictions based entirely on the top scoring pairs. In the case of IPS study, there is in fact a unique top scoring pair - HGFAC and APCS, which appears to have biological interest. However, there may be many other pairs whose relative expression values are informative. One possible direction of future work is to find a more stable, comparison-based signature combining multiple high scoring pairs. For example, one may envision a ProtPair classification based on all protein pairs achieving the k best scores. In this case, k is a parameter whose optimal value can be estimated using cross-validation.

An alternative discovery based approach to using ProtPair would be to use it to detect variation in abundance patterns in comprehensive peptide lists, irrespective of their annotation status. For example, Rosetta Elucidator framework was used to extract raw peak areas from SICs corresponding to all of the 11108 isotopic features observed in MS1, regardless of the presence/annotation of the tandem MS spectrum for the feature. Note that confident annotation MS/MS is available for only 1799 peptides of the overall 11108 features. Using pairwise comparison of these comprehensive list of global features led to the best discriminating feature pair shown in Figure 5, where the 2 axes mark the abundances of individual features. The figure reveals a clear separation between the 2 groups as defined by the feature intensities. Due to the low signal intensity of such features, it is likely that they are typically either not selected for tandem MS or had extremely low signal in the tandem MS leading to missed/low confidence assignment. However, the MS1 data could be used in an alternative targeted MS approach, which is specially useful to target low abundance but important proteins/peptides that may experience a negative bias towards selection during the tandem MS sampling. Such integration between experimental and computational workflows can be very valuable for targeted MS in the future.

The results already provide evidence that discriminating comparisons among protein expression levels can be discovered even under conditions of small sample size. Given the large number of variables (peptides/proteins), a patient population sample size of 21 is considered small. With number of samples in the order of hundreds, more complex decision trees can be learned from the data, using only comparison questions, thus maintaining easily interpretable results that do not require any normalization. The corresponding decision rules would then be based on more complex peptide abundance comparisons involving more than two proteins. The methodology can also be extended to more complex and heterogeneous data sets, for example those combined from samples obtained from various sources such as plasma, urine, or organ specific tissue as well as other MS variables that reflect intensity, such as spectral counts. With small amounts of data, it may only be possible to collect reliable estimates of pairwise comparisons among expression levels so as to avoid overfitting. More data could be used to model the statistical dependency structure among families of proteins such as metabolic and regulatory pathways etc. This approach lends itself to a natural, hierarchical family of models which can accommodate various kinds and amounts of data.

## Conclusions

A methodology to molecular classification for disease progression using pairs of peptides/proteins from shotgun mass spectrometry based proteomics data has been presented. The strength of this linear approach lies in its design for being able to handle high dimensional data with small sample size by (i) using minimal number of features in order to avoid over fitting of the data (ii) observing consistency of differential expression change across two disease conditions (iii) aggregating peptide pairs originating from the same pair of proteins to classify at the level of protein pairs through joint statistics. Since proteins act as the true functional machines in an organism, predictions based upon ratios of protein expression levels provide a natural link with biological phenomenon. The method has been tested using clinical proteomics data from 21 patients following a bone marrow transplant, differentiating between 13 patients progressing to IPS versus 8 controls that remain unaffected by IPS. The approach gains power by combining multiple peptide pairs originating from the same set of proteins, with each unique peptide pair providing an independent measure of discriminatory power. The prediction rate of the ProtPair for IPS study as measured by leave-one-out CV is 69.1%, which may be very beneficial for clinical diagnosis as it may flag patients in need of more frequent monitoring. It was encouraging to find that the “top ranked” proteins provided by ProtPair are known to be associated with the biological processes and pathways intimately associated with known IPS biology based on both mouse models, such as respiratory disease, inflammatory responses, and acute phase response. Proteins from top 20 pairs were imported into IPA to see if they shared common biological networks. The network was found to be enriched in acute phase response proteins whose concentration is known to change significantly in response to inflammation, and is associated with respiratory disorders, hematological dysfunction, cardiovascular complications, and infectious diseases. The data indicates that patients with improper regulation in the concentration of specific acute phase response proteins at the time of bone marrow transplant are highly likely to develop IPS within few weeks. The results lead to a specific set of protein pairs that can be efficiently verified by investigating the pairwise abundance change in independent cohorts using ELISA or targeted mass spectrometry techniques.

## Competing interests

The authors declare that they have no competing interests.

## Author’s contributions

PK designed and implemented the ProtPair Classifier; PK and MRC drafted the manuscript; DS performed the MS experiments. MRC and KC designed the overall IPS study; MRC participated in the design and co-ordination of the study. All authors read and approved the final manuscript.
